# Intraspecific Variation in Nectar Chemistry and Its Implications for Insect Visitors: The Case of the Medicinal Plant, *Polemonium Caeruleum* L.

**DOI:** 10.3390/plants9101297

**Published:** 2020-10-01

**Authors:** Justyna Ryniewicz, Mateusz Skłodowski, Magdalena Chmur, Andrzej Bajguz, Katarzyna Roguz, Agata Roguz, Marcin Zych

**Affiliations:** 1 Botanic Garden, Faculty of Biology, University of Warsaw, 00-478 Warsaw, Poland; m.sklodowski@biol.uw.edu.pl (M.S.); k.roguz@biol.uw.edu.pl (K.R.); 2 Department of Biology and Ecology of Plants, Faculty of Biology, University of Bialystok, 15-245 Bialystok, Poland; m.chmur@uwb.edu.pl (M.C.); abajguz@uwb.edu.pl (A.B.); 3 Feature Forest, Trzy Lipy 3, 80-172 Gdańsk, Poland; agata_roguz@o2.pl

**Keywords:** nectar composition, pollination, reproductive ecology, variation in plant traits, generalist species

## Abstract

Floral nectar, being a primary reward for insect visitors, is a key factor in shaping plant–pollinator interactions. However, little is known about the variability in nectar traits, which could potentially affect pollinators and the reproduction of the species. We investigated intraspecific variation in nectar traits in 14 populations of a Red-listed plant*, Polemonium caeruleum*. Populations varied in terms of the proportion of self-compatible and self-incompatible individuals, and insect communities visiting flowers. Using HPLC, we determined the nectar sugar and amino acid (AA) composition and concentration. We also recorded some basic habitat parameters, which could influence nectar chemistry. In seven selected populations, we investigated the taxonomic composition of the insects visiting flowers. Our observations revealed significant intraspecific variability in nectar chemistry in *P. caeruleum*. Nectar production was male-biased, with male-phase flowers secreting sucrose- and AA-rich nectar. An analysis revealed that variability in *P. caeruleum* nectar may be slightly shaped by environmental factors. The studied nectar characters, especially sugars, had little effect on insects visiting flowers. We argue that variation in nectar traits in this generalist plant is a matter of random genetic drift or “adaptive wandering” rather than directional specialization and adaptation in the most effective and abundant group of pollinators.

## 1. Introduction

Nectar, being primarily a sugar solution, has been perceived as the most crucial floral food reward for pollinators [1]. However, recent studies have demonstrated that because of the presence of many non-sugar constituents, such as amino acids (AAs), phenolic compounds, or alkaloids, floral nectar is much more than just food. Its functions extend beyond its relationships with pollinators [2,3,4,5,6]. Therefore, it should be regarded as a complicated multifunctional interface between plants, and their mutualists and antagonists. Since nectar production is under pollinator-mediated selection, parameters such as composition, volume, and sugar concentration may be highly variable between plant species [1,7,8]. Earlier studies of nectar diversity have postulated that some nectar features, such as sugar and AA profiles, are species-invariant [7,9,10,11], and some new analyses support a rather conservative proportion of nectar components, that is sugars, between populations of the same species (e.g., [12]). However, this notion seems to be the result of technical difficulties associated with early analytical methods [3,13] because many recent studies have reported variation in nectar traits between populations, within populations, within one inflorescence, or even among sexual phases of the same flower [8,12,13,14,15,16,17,18,19,20]. Such variations are mainly a consequence of environmental factors, such as soil properties, air temperature, and sun exposure [1,2,8,13,21], and, according to the latest study, microorganisms inhabiting nectar [22,23]. Importantly, the landscape-scale variability in floral rewards may be correlated with habitat quality and may have a significant effect on the reproductive success of a population [13,17,18,20]. Unfortunately, this variation in nectar constituents, especially in nectar AAs, is still gravely understudied and poorly understood.

In our study, we chose *Polemonium caeruleum* L. (Polemoniaceae), which produces copious, sucrose-dominant nectar that is rich in proline [24]. However, this information is based on the sampling of a single population, and recently published data [25], and our observations suggest substantial geographic variations in the breeding system and pollinator assemblages (dominance of bees vs. flies) for this plant species. As recently reported, such differences also include spatial variation in the bacterial microbiome in the nectar of *P. caeruleum* [26]. In the context of the aforementioned problems, we expected differentiation during the study of nectar traits among 14 populations of *P. caeruleum*. We also determined whether, and to what extent, nectar features are influenced by habitat, and whether the observed variation in pollinator assemblages is associated with differences in nectar chemistry among *P. caeruleum* populations, because many pollinators exhibit specific preferences for some of the constituents [6,8,27]. Improving our knowledge concerning the biology, ecology, and intraspecific diversity of this species increases the potential for more efficient protection.

## 2. Results

### 2.1. Sugars

**Figure 1:**
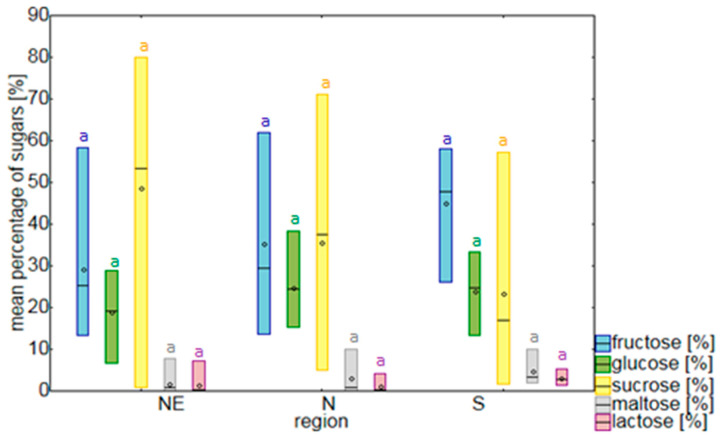
Mean percentage of nectar sugars in populations of *Polemonium caeruleum* from three regions in Poland. The sample number for each region was, respectively, *n* = 18 for the northeastern (NE) populations, *n* = 7 for the northern (N) populations, and *n* = 4 for the southern (S) populations. The line in the middle of the box represents the median and the circle represents the mean. Boxes extend from the 5th to 95th percentiles. There were no significant differences in sugar percentages between regions (*p* > 0.05).

The mean nectar sugar concentration for the investigated populations of *P. caeruleum* was 409.9 ± 344.8 µg/µL (range was from 145.72 to 1102.7 µg/µL per population), with significant differences between the studied populations (Chi-square = 22.87, df = 13, *p* = 0.04) (Table 1). The main sugar components detected in all study samples were sucrose, fructose, and glucose (with mean proportions, respectively, of 42.1 ± 22.7%, 32.8 ± 14.4%, and 21.0 ± 7.1%). Maltose and lactose were also present in most of the analyzed samples (characterized by mean proportions, respectively, of 2.5 ± 3.2 and 1.6 ± 2.1%). The proportions of detected sugars were highly variable among the studied populations, with significant differences in sugar composition between populations for maltose (Chi-square = 24.71, df = 13, *p* = 0.03). Considering the geographical distribution of populations, sucrose predominated in northeastern Poland, whereas fructose predominated in the southern populations. However, these differences were not significant (Figure 1). There were also no differences in sugar concentration between the three designated geographical regions representing groups of studied populations (Chi-square = 10.75, df = 2, *p* > 0.05).

Similar values of total sugar content (µg/µL) in nectar samples were observed during male and female sexual phases, however there were significant differences in individual sugar proportions between the two phases (Table 2). The percentage of glucose and sucrose, as well as the sucrose/hexose ratio, varied significantly (*p*-values were, respectively, 0.02, 0.04, and 0.03). In the male phase, during the early flowering stage, sucrose was dominant (with a mean proportion of 50.34 ± 22.3%), whereas in the later female phase, hexoses represented a higher percentage (with a mean proportion of 61.93 ± 18.05%). Only one population (MAL) was characterized by hexose dominance, wherein the above gender-biased proportions were reversed.

### 2.2. Amino Acids

We detected 29 AAs in nectar samples of *P. caeruleum* (ranging from 19 to 27 AAs per population). The mean AA concentration was 590.3 ± 1006 pmol/µL (ranging from 110.2 to 3416.3 pmol/µL; Supplementary Materials, Table S1). We did not detect significant differences in total AA concentration between populations (Chi-square = 20.50, df = 13, *p* > 0.05; Table 1). Among the detected AAs, all 20 protein AAs (PAAs) and nine non-protein AAs (NPAA) were present, with a mean proportion of 48:11. Among PAAs, the highest percentage was noted for glutamine, (15.8 ± 10.4), glutamic acid (14.5 ± 4.8), and serine (9.0 ± 3.1). Among NPAAs, the highest percentage was that of β-Alanine (BALA; 8.1 ± 3.2%), β-aminobutyric acid (BABA; 4.7 ± 4.5%), and ornithine (3.1 ± 2.6). We determined the proportions of essential amino acids (EAAs) for honeybees (as well as other pollinators), which were phenylalanine, leucine, arginine, threonine, lysine, isoleucine, valine, methionine, histidine, and tryptophan [28,29]. All 10 EAAs were found in nectar samples, and phenylalanine had the highest share of 3.9 ± 2.2% among them. The proportions of EAAs varied significantly between populations (from 8.4% to 39.5%; F = 5.89, df = 13, *p* = 0.0007), with a mean value for all populations of 19.5 ± 8.5% (Table 1). γ-aminobutyric acid (GABA) was the rarest AA, being present in only one population. Proline, the AA characterized by the highest share in a previous study [24], was found in six populations at a rather low proportion (mean of 1.7 ± 4.0%). Total AA concentration was almost two times higher in the male than the female flowering phase (although this was not significant; *p* > 0.05; Table 2).

For the linear mixed models (LMM), we used the percentage of six AAs. Glutamine, glutamic acid, and serine were selected because they had the highest proportion in nectar samples (means of 15.8 ± 10.4%, 14.5 ± 4.8%, and 9.0 ± 3.1%, respectively). Isoleucine, phenylalanine, and norvaline were selected, using the RFE method, as having the strongest positive influence on the frequency of visits by insects. For the AAs used in the LMM, there were significant differences between populations in three out of six AAs: glutamine (F = 7.84, df = 13, *p* < 0.001), glutamic acid (F = 6.52, df = 13, *p* < 0.001), and phenylalanine (F = 9.35, df = 13, *p* < 0.001).

**Figure 2:**
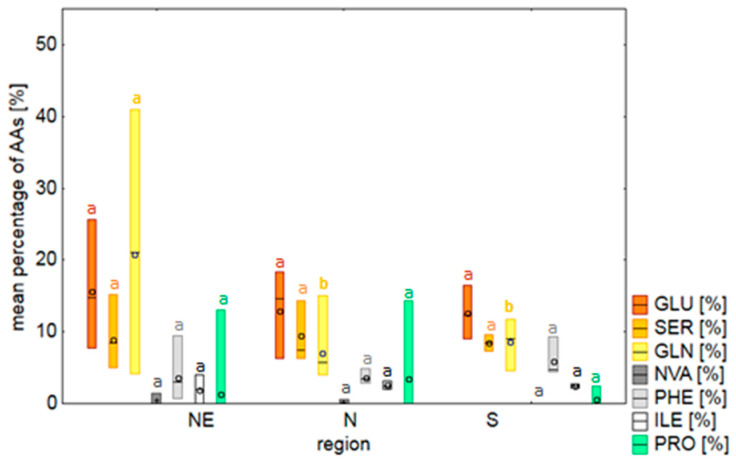
The mean percentage of AAs selected for the linear mixed models and proline (vital AA for pollinators) in populations of *Polemonium caeruleum* from three regions in Poland. GLN (glutamine), GLU (glutamic acid), SER (serine); ILE (isoleucine), PHE (phenylalanine), NVA (norvaline), and PRO (proline). The sample number for each region was *n* = 18 for northeastern (NE) populations, *n* = 7 for northern (*n*) populations, and *n* = 4 for southern (S) populations. The line in the middle of the box represents the median and the circle represents the mean. Boxes extend from the 5th to 95th percentiles. Means of AAs that varied significantly between regions are marked with different letters (*p* < 0.05).

**Figure 3:**
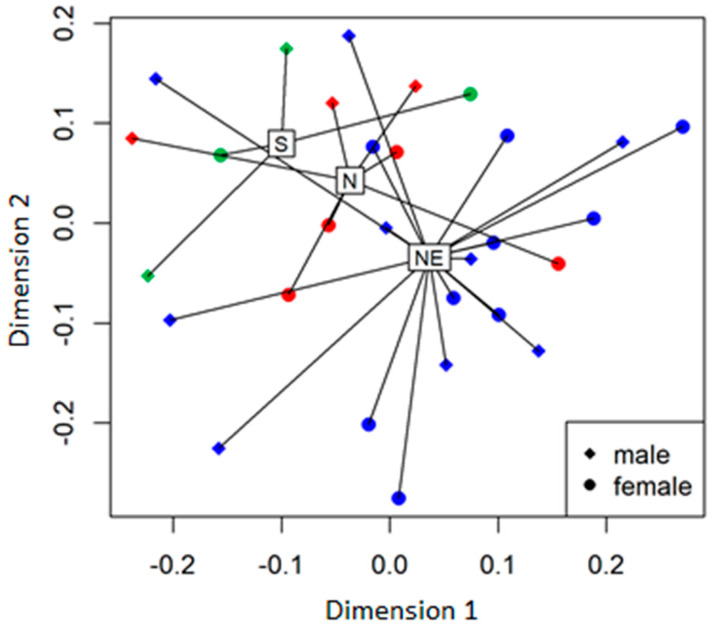
Non-metric multidimensional scaling (NMDS) results based on Bray–Curtis distances of nectar component proportions (sugars and AAs) of *Polemonium caeruleum* during the male and female flowering phase from 14 populations. Colors indicate groupings by regions, which are significantly different: northern (N)—red, northeastern (NE)—blue, and southern (S)—green (F = 2.34, df = 2, *p* = 0.04). Each sample is displayed as a square (for the male flowering phase) and a circle (for the female flowering phase).

When the geographic affinity of populations was studied (Figure 2), we found that the northeastern populations were characterized by a higher percentage of glutamine than the northern and southern populations (F = 9.15, df = 2, *p* = 0.001), whereas the proportions of other analyzed AAs were not significantly different (*p* > 0.05). There were also no differences in AA concentrations between the three regions (Chi-square = 9.24, df = 2, *p* > 0.05). Additionally, the SPN population, which in previous years (unpublished data) was distinguished from other populations by the predominance of flies as the main flower visitors, was characterized by the highest percentage of BABA, BALA, tyrosine, and valine. NMDS analysis revealed that the proportions of detected sugars and AAs group by region are significantly different (Figure 3).

### 2.3. Biomass and Soil Analysis

We found significant differences between populations in total N (F = 4.83, df = 13, *p* = 0.003) content in biomass, and Fe (F = 25.57, df = 13, *p* < 0.001) and Ca (F = 27.70, df = 13, *p* < 0.001) in soil (see Supplementary Materials: Table S2).

The LMMs showed that the basic habitat parameters measured influenced nectar traits for only three out of 13 analyzed nectar characters (Table 3). The total AA content was positively influenced by Fe in the soil. There was a negative relationship between serine and Ca in the soil. Moreover, total K content in biomass negatively affected the percentage of norvaline and serine in nectar, whereas total P content in biomass positively influenced the percentage of norvaline and negatively affected the percentage of serine in the nectar.

### 2.4. Taxonomic Composition and the Frequency of Insect Visits in the Selected Populations

**Figure 4:**
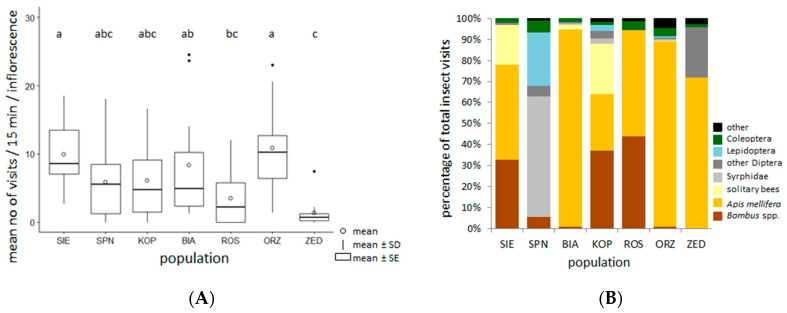
(**A**) Mean frequency of insect visits in seven selected populations of *Polemonium caeruleum*. Means with various letters are different at *p* < 0.05. (**B**) Taxonomic diversity of insects visiting flowers. Populations are arranged in ascending order according to their size.

We recorded 1835 insect visits to *P. caeruleum* flowers in seven studied populations, and the overall visit frequency was 6.5 ± 6.2 visits per census (15 min) per inflorescence. We observed significant differences in the frequency of visits between populations (Chi-square = 27.64, df = 6, *p* < 0.001; Figure 4A), with the highest frequency in ORZ and the lowest in ZED. Flowers of *P. caeruleum* were most often visited by insects represented by four taxonomic orders: Hymenoptera, Diptera, Lepidoptera, and Coleoptera. However, representatives of the Hymenoptera order were the most abundant visitors in all but one study population, and all included honeybees (*Apis mellifera*, 60.2% of visits), bumblebees (17.3%), and solitary bees (9.7%) (Figure 4B). Some of the populations (BIA, ORZ, and ZED) were strongly dominated by honeybee visits. Dipterans (Syrphidae and other flies) were the second most abundant taxon after the order Hymenoptera and were responsible for 4.2% of all visits. The SPN population was distinguished by the domination of insects by the order Diptera, whereas bees were responsible for only 5.7% of all visits (Figure 4B).

The noted frequency of visits was influenced by the presence of AAs in *p. caeruleum* nectar, with different AAs influencing the recorded groups of visitors. The frequency of Syphidae and Lepidoptera was negatively correlated with total AA concentration. We also detected several negative correlations between a specific AA and pollinator group; for example, there was a negative correlation between visits by *Bombus* spp. and tryptophan presence. According to our data, the highest number of correlations with AAs was with Lepidoptera (Table 4).

## 3. Discussion

### 3.1. Nectar Sugars

The mean recorded sugar concentration in *P. caeruleum* nectar was 398.8 ± 345.9 µg/µL and it varied significantly between populations. Similarly to other plant species, the nectar of *P. caeruleum* is composed mainly of sucrose, fructose, and glucose [1,6,10,21]. Sucrose was the dominant carbohydrate in the majority of the analyzed populations, followed by fructose and glucose. This agrees with earlier results for *P. caeruleum* [24], as well as most of the other representatives of Polemoniaceae [30]. However, this situation was not universal because, in some of the studied populations (MAL, BIA), the sucrose content was relatively low (from 5.5% to 20%). According to the classification of Baker and Baker [7], the floral nectar of *P. caeruleum* in half of the study population was sucrose-dominant, whereas the others were sucrose-rich, hexose-rich, and one was even hexose-dominant. Maltose and lactose, which were not detected in earlier studies on *P. caeruleum* nectar [24], were also present in 10 of the 14 study populations, reaching quite high concentrations in some populations. Despite differences among the examined sugar traits between populations, we did not observe any trends for the three designated geographical regions.

Considerable inter-population variability in nectar sugar concentration and composition has been already reported for other plant species [13,14,31,32]; however, the knowledge concerning factors shaping the variability of nectar sugars requires supplementation. Sugar content and composition can be affected by nutrients in the habitat [33,34]; however, this study did not confirm such an influence, at least where nectar sugars are concerned.

The sugar profile of *P. caeruleum* nectar may be highly attractive to a diversity of groups of potential pollinators. The level of sugar concentration present in the nectar of *P. caeruleum* is close to the average value characteristic for plants visited by bees and flies [1,35]. Moreover, bumblebees and honeybees, the main visitors of *P. caeruleum* flowers, as well as other hymenopterans, butterflies, and moths, prefer sucrose over fructose and glucose [28,29,35,36,37]. On the other hand, nectar in populations characterized by higher proportions of hexoses over sucrose may be attractive to less specialized pollinator guilds, for example, flies [21]. The presence of two disaccharides, maltose and lactose, which were not previously detected in *P. caeruleum* nectar, is quite a surprising result, because maltose and lactose are regarded as less attractive and less nutritionally useful for pollinators [38]. To our knowledge, information regarding their role is lacking, and these sugars are often overlooked in analyses.

The relationship between flower visitor assemblages and the nectar sugar profile has been confirmed by other authors [31,39,40]; however, we did not find strong connections between nectar sugar composition and concentration and the frequency of visits by a specific group of insects. Only the percentage share of glucose was positively correlated with the two affected insect groups: butterflies and hoverflies, with the latter being known for their preference for this sugar [21]. Little evidence of a relationship between nectar sugar characteristics and the frequency of insect visits (as well as that of a particular insect group) appeared to confirm the generalist pollination strategy of the investigated species. Moreover, because *P. caeruleum* is characterized by a rather small number of ovules (mean 29 [24]), even less specialized pollinators (such as flies) may provide a sufficient amount of pollen for adequate pollination. In this case, plant specialization for a particular taxonomic group of pollinators may be an unprofitable strategy (see [12]). Furthermore, high fluctuations in the number of individuals in *P. caeruleum* populations can lead to random variation, which may result in a high diversity of nectar characteristics. The high fruiting level and sporadic pollen limitation in *P. caeruleum* populations (Ryniewicz et al., unpublished data) may support this assumption.

Our results revealed significant differences in the proportion of sucrose and hexoses between floral sexual phases of *P. caeruleum*; however, our results were the opposite of that previously reported [24]. In the female phase, the participation of sucrose significantly decreased, whereas participation of both hexoses increased in comparison to the male phase. This could be caused by flower aging and consequent hydrolysis of sucrose into hexoses [19,41,42,43]. Gender-biased nectar composition could also be maintained by sexual selection or inbreeding avoidance, which is a common feature, especially for plants with dichogamous or heterostylous flowers [44]. A male-biased sucrose share may be an adaptation to enhance pollinator frequency to flowers because male fitness is most strongly limited by access to mates [45].

### 3.2. Amino Acids

Our analysis revealed that the mean content of AAs in nectar samples was 590.3 ± 1006 pmol/µL and it did not differ among populations. However, despite the lack of significant differences in AA concentration, we found significant variation in nectar AA composition between study populations (Table 1, Supplementary Materials Table S3), as has been presented for other plant species [12,13,14,20,46]. On the other hand, when considering the differences between the three designated geographical regions, there were no observed trends in the AA profile, despite that the percentage share of glutamine varied significantly.

As with sugars, the presence of AAs in floral nectar may be shaped by the availability of nutrients in the habitat [42]. For example, it was shown that the total AA concentration might be influenced by N in the soil [13,18,47]. However, according to our analyses, AA concentration was not affected by N but by the Fe content in the soil. This element plays an important role in various physiological and biochemical pathways [48]. Nonetheless, its influence on the concentration of AAs in floral nectar has not been previously confirmed. Our results also revealed the effect of the studied macro- and microelements on certain AAs, which opens a new field for further study.

The values representing the AA concentration in *P. caeruleum* nectar were similar to those of sugars, and are regarded as preferred by flies (560 pmol/µL) and bees (620 pmol/µL) [10], the two main insect orders visiting flowers of this plant. Most of the 29 AAs that were present in the nectar of *P. caeruleum* often occur in the nectar of other plant species [13,15,42,49,50]. In addition to AAs common in floral nectar, our analysis also revealed the presence of sarcosine, tryptophan, and norvaline, which have been rarely reported in earlier analyses [20,50,51]. However, even the effect of well-known AAs on pollinators is often not known. Among the AAs characterized by the highest percentage in the nectar of *P. caeruleum*, glutamine is considered a beneficial addition to the energetically expensive process of flight in insects [47]. Glutamic acid and serine may influence the growth and reproduction of insects [52], and glutamate (the anion of glutamic acid) plays an essential role in the nervous system of insects in regards to learning and memory (reviewed in [53]). Isoleucine, phenylalanine, and norvaline were selected for the LMM model as the most influential on the frequency of insect visits. Isoleucine and phenylalanine belong to EAAs [54,55] and are among AAs that stimulate the sugar cell receptors of insects [56]. Phenylalanine is also considered a phagostimulator for bees [57]. The functions of norvaline, which have already been recorded in the nectar of some monocotyledons [20,50], remain unclear.

Although non-protein AAs (NPAAs) often reach a high percentage among the AAs in floral nectar, their ecological function is poorly understood. However, studies have shown that BALA, the most common NPAA in our study, is abundant in the insect visual system [58], and can also be involved in many behaviors influencing foraging, such as learning and memory [59].

Earlier studies conducted by Zych [24] revealed that the AA proline plays a pivotal role in insects, including serving as an energy source, [60], and was characterized by the highest share among all AAs present in the nectar of *P. caeruleum*. In contrast, according to our results, proline was present in nectar collected from less than a half of the populations, only from male-phase flowers, and was characterized by a rather low concentration (from 0 to 6.5%). Furthermore, proline was absent in samples obtained from a population that was previously studied. This indicated high variability in nectar composition in subsequent years.

According to Gijbels [13], the frequency of insects visiting flowers is affected not by the total content of AAs, but by the presence and content of individual AAs. Considering the relationships between nectar AA composition or concentration and the frequency of visits by a particular group of insects, we reported several dependencies. The frequency of visits by bumblebees was negatively affected by tryptophan and lysine. As already reported, tryptophan may be repellent for bees [42], and to the best of our knowledge, this effect has not been described for lysine. Norvaline, a rarely detected AA in floral nectar, had a positive effect on the frequency of visits by honeybees, and glycine (AA that contributes to insect growth [52]) positively influenced the frequency of visits by hoverflies.

Our results also suggest the influence of certain AAs on the frequency of butterfly visits. This parameter was positively correlated with the percentage of alanine, tyrosine, valine, and BABA and negatively correlated with AABA. Of these amino acids, in the context of their effect on butterflies, the most is known about tyrosine, which is the initial precursor for pigments among those insects [61]. Additionally, for both butterflies and hoverflies, we noted a negative relationship between the frequency of visits to flowers and total AA concentration. Higher concentrations of AAs in nectar are especially important for adult butterflies because it is the only source of nitrogen [62], however, these insects were responsible for just over 1% of visits and rarely exhibited flower constancy [63]. Syrphids, on the other hand, feed mainly on pollen [64].

Variation in nectar AA composition between populations of *P. caeruleum*, similar to that of sugars, is more a matter of random changes than deliberate specialization and adaptation to the most effective group of pollinators. However, a diverse AA composition and concentration may be an incentive for visits by diverse groups of insects characterized by different AA preferences, because AAs contribute to the taste of nectar [55,63] and its scent [65].

As in the case of sucrose, the mean AA concentration was almost two times greater during the male than the female sexual stage, which is probably also associated with increasing the expenditure of the plant on attractiveness during the male flower phase.

### 3.3. Other Factors that May Have Affected Nectar Properties and Mutualistic Interactions with Insects

In addition to the factors analyzed here, several other agents may affect the nectar characteristics, for example, the microclimate specific for each population [14]. These factors include water availability [1], ambient humidity [66], temperature, or sunlight exposure [15].

Different weather conditions in populations localized in different Polish regions may probably result in dissimilar effects on various nectar characteristics. For example, high ambient temperatures during nectar collection in the two largest populations (ZED and ORZ) probably resulted in extremely high sugar concentrations (above 1000 µg/µL). Nectar concentration is associated with high viscosity, thus restricting its use by some insects, especially those with long tongues [1]. It may lower their visitation rate, for example, the ZED population is characterized by the highest sugar content in nectar and the lowest visitation rate. Finally, we observed that some floral visitors collected only pollen, which could also affect the relationship between nectar characters and potential pollinators.

Besides weather conditions, population size could be one of the factors influencing the relationship between nectar characters and insect visits. Fluctuations in subsequent years regarding the number of flowering shoots in some of the studied populations also contributed to the random fixation of nectar features.

Each of the study populations was characterized by different local conditions, affecting the composition of assemblages of potential pollinators. This is associated with the food base, nesting places, and other factors that may shape insect communities. The presence of apiaries in the vicinity of most of the studied populations of *P. caeruleum* and the introduced honeybees largely shaped the share and frequency of insect visits to flowers. Two populations localized in national parks (KOP and SPN) were characterized by low or a lack of participation by honeybees among the floral visitors.

Insects visiting flowers are also among the factors that may affect the variability of studied nectar traits between populations. They may influence nectar volume [41], concentration, and composition [31], or may transfer microorganisms affecting nectar properties [23,67,68]. Bacteria and yeast inhabiting nectar may influence sugar and AA composition, pH, volume, and emitted volatiles, modifying plant–pollinator interactions [23,68,69,70]. Since we sampled nectar from flowers that were previously available for pollinators, there is a high probability that microorganisms inhabited it. A study in which two of the populations analyzed here were included demonstrated that bacterial communities in the nectar of *P. caeruleum* varied between populations [26]. When considering the proportion of sugars in the male and female flowering phases, the metabolic activity of microorganisms may increase the hexose concentration in the female phase [43,71]. Microorganisms inhabiting nectar may also affect the nectar AA profile. Vannette and Fukami [23] found that inoculation of wildflower nectar with bacterial strains increased, whereas inoculation with yeast decreased AA concentration. Therefore, changes in the AA profile in the nectar may also be the result of the activity of microorganisms; however, this awaits further study.

Our analysis revealed the high differentiation of analyzed nectar traits between populations of our model species. The measured habitat parameters, such as habitat fertility and soil properties, that could influence nectar chemistry did not affect it to a high extent. The relatively small effect of insects visiting flowers on nectar characters indicates that shaping them is a matter of random genetic drift or “adaptive wandering” than directional specialization and adaptation in the most effective and abundant group of pollinators.

## 4. Conclusions

In this study, we analyzed variations in nectar traits of a generalist plant*, P. caeruleum*, their causes, and consequences. Our analyses revealed two sugars and 19 AAs that were not previously detected in *P. caeruleum* nectar. Some of the investigated features of the nectar profile, including sugars and AAs, are variable among 14 populations of our model plant. When considering three geographic regions, in which the populations are clustered, analyses revealed that the proportions of detected sugars and AAs group by region are significantly different. We also detected differences in sugar proportions during the male and female sexual stages of flowering. The male phase is characterized by a significantly higher proportion of sucrose, which may be an adaptation to enhance pollinator frequency on flowers.

Our results indicate that the habitat may play a role in shaping nectar features. On the other hand, analyzed nectar features had little effect on insects visiting flowers, which supports the hypothesis that, regarding this species, nectar characteristics are a consequence of random genetic changes.

There is still a need to supplement knowledge concerning inter-population differences in nectar characters, especially those involving generalist plant species. Studies on the biology of *P. caeruleum*, including factors potentially affecting its reproductive success, are essential for proposing an effective strategy for the protection of this species that disappears in Poland. Our results suggest that the nectar of *P. caeruleum* may be influenced by macro- and microelements in the habitat, and it is highly probable that intensive fertilization of land adjacent to populations may result in changes in nectar composition, including an increase in the proportion of AAs repellent for pollinators. This, however, awaits further study, which should also include other factors that may affect this floral reward, not considered in this study (e.g., historical and environmental). Recommendations for the protection of *P. caeruleum* populations should focus on habitat protection, as land drainage probably leads to lower nectar production, consequently reducing its attractiveness to insects.

## 5. Materials and Methods

### 5.1. Plant Description

*Polemonium caeruleum* L. (Jacob’s ladder; Polemoniaceae) is a perennial, boreal herb usually associated with wetlands and damp meadows in temperate regions of the Northern Hemisphere [72]. It is a medicinal plant containing many active substances, including those with antibacterial and antitrypanosomal properties [73]. In Poland, the plant is included in the Polish Red Book of Plants (VU category). Land-use change and water drainage [74] as well as a decrease in the number of pollinators [24] have negatively affected the number and size of *P. caeruleum* populations in Poland.

Inflorescences of *P. caeruleum* consist of a few to over a dozen simultaneously opened flowers. Campanulate flowers with light blue to violet corollas are usually protandrous and gather in a corymbose inflorescence [75]. Nectar is secreted at the base of the ovary and accumulates in the corolla tube where it is protected by hairs [76]. As reported for one of the Polish populations, nectar is sucrose-dominant, proline-rich, and female-biased, as the female phase is characterized by higher nectar production and concentration. The male phase is shorter than that of females and lasts on average 1.7 ± 0.9 d vs. female 2.0 ± 0.8 d [24]. Additionally, nectar sugar concentration appears to be highly variable with single flowers producing from 1.07 with a concentration of 16.5% [24] to 3.16 mg of nectar per flower with a concentration of 41.8% [77].

The plant reproduces only by seeds [72] and is visited by a broad spectrum of insects collecting nectar and pollen, indicating a generalist pollination system [24]. The most effective pollinators are social bees, which are responsible for 70–91% of pollination [24,25]. Observations from Poland showed that populations of *P. caeruleum* are characterized by a mixed-mating system [25], with plants producing seeds via both self- and cross-fertilization.

### 5.2. Field Observations

**Figure 5:**
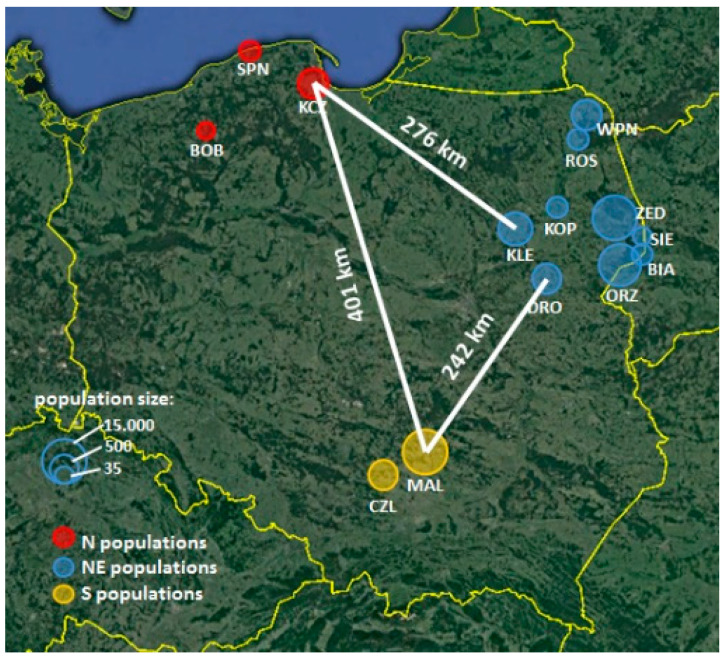
Distribution of *Polemonium caeruleum* study populations in Poland. The size of the circles corresponds to the log-transformed size of populations (number of flowering shoots), and the circle color corresponds to one of the three regions. The distances between the closest populations from each region are presented.

We focused on 14 populations of *P. caeruleum,* across the entire Polish range for the species, varying in size (both in terms of the number of individuals and occupied area), distribution, and types of plant communities in their immediate vicinity (Figure 5; Supplementary Materials: Table S1). Since our methodology required heavy equipment, in this study we included all Polish populations of *P. caeruleum* known from the literature [78,79,80,81], and personal communications that were within walking distance (≤2 km) from roads accessible by 4WD vehicles. In 2018, during the peak of the flowering period (June in Poland), we investigated nectar properties and basic habitat parameters. Due to the time constraints and that the flowering period in most of the study populations overlapped, we analyzed the taxonomic composition and frequency of insects visiting flowers in seven populations. We selected populations that were relatively close to each other and characterized by variable sizes. Additionally, we included the SPN population, distinguished from others by the predominance of flies as the main flower visitors in previous study years (unpublished data). According to geographical localization, populations were clustered in three regions, including the northern (BOB, SPN, and KCZ), northeastern (WPN, ROS, KOP, ZED, SIE, BIA, ORZ, DRO, and KLE), and southern (MAL and CZL) populations, in which the distance between populations was between 23 and 178 km.

### 5.3. Nectar Sampling and Analysing

To analyze nectar sugar and AA concentration and composition, 24 h before nectar sampling in each of the 14 populations, we randomly selected 10–20 inflorescences (depending on population size) at full bloom and bagged them with fine nylon mesh (to prevent the removal of nectar by insects). Samples were collected from all available flowers (usually several from one inflorescence), using a pipette and sterile tips, and stored in Eppendorf tubes until further analysis. We collected nectar separately from flowers in male (M) and female (F) sexual stages, which resulted in two samples obtained from each population (except the BOB population, where we collected three samples: two for male flowers and one for female flowers). Due to the small amount of nectar in a single *P. caeruleum* flower, each sample consisted of nectar obtained from 20–30 flowers. After collection, nectar samples were stored at 4 °C in a portable cooler until being moved to the lab, and then were frozen at −20 °C and further analyzed. Nectar chemistry (sugar and AA content and composition) was analyzed using high-performance liquid chromatography (HPLC) [50,82] at the University of Białystok.

Nectar samples were diluted with distilled water (for sugar analysis, 10 µL of nectar + 40 µL of water and for AAs analysis, 10 μL of nectar and 10 μL of water). Samples were filtered through spin columns using a 0.4 µm pore size membrane filter before injection, and the supernatant was loaded into the insert and analyzed by HPLC. The samples were analyzed using an Agilent Technologies 1260 Infinity series system consisting of a 1260 Infinity Agilent Quaternary pump G1311B, a 1260 Infinity Diode Array Detector (DAD) G1315D, a 1260 Infinity Fluorescence Detector (FLD) G1321B, a 1260 Infinity ALS G1329B Automated Sample Injector, a 1290 Infinity Autosampler Thermostat G1330B, and an 1290 Infinity TCC G1316C thermostatted column oven. The system was controlled using the Agilent OpenLab ChemStation software.

For sugar analysis, we used a Zorbax Carbohydrate Analysis Column (4.6 mm × 250 mm, 5 µm). A 10 µL aliquot sample or standard solution was injected. The separation was conducted at 30 °C with a mobile phase comprising acetonitrile/water (70:30, v/v) at a flow rate of 1.4 mL/min. The analytical data were integrated using the Agilent OpenLab CDS ChemStation software for liquid chromatography (LC) systems. Sugars (glucose, fructose, sucrose, maltose, and lactose) were identified based on comparisons of peak areas obtained for the collected samples with those of the reference solutions [82].

The analysis of AAs in 10 μL aliquots of nectar collected from flowers was performed by gradient HPLC using an Agilent Zorbax Eclipse Plus C18 (4.6 × 150 mm, 5 μm) column with a guard. The extracts containing primary and secondary AAs were pre-column-derivatized with the *o*-phthalaldehyde (OPA) and 9-fluorenylmethyl chloroformate (FMOC) reagents. An injector program was used for the derivatization. Following the derivatization, a mixture of each sample was injected into a pre-equilibrated column operating at 40 °C. The primary (OPA-derivatized) AAs were monitored at 388 nm by DAD, whereas the secondary (FMOC-derivatized) AAs were monitored by FLD at an excitation wavelength of 266 nm and an emission wavelength of 305 nm. Mobile phase A was 40 mM NaH_2_PO_4_ (pH 7.8, adjusted using 10 M NaOH solution), whereas mobile phase B was acetonitrile/methanol/water (45:45:10. v/v/v). The following gradient profile was observed: 0–5 min, 0–10% B; 5–25 min, 10–40.5% B, 25–30 min, 40.5–63% B; 30–35 min, 63–82% B; 35–37 min, 82–100 B; 37–39 min, 100% B; 39–40 min, 100% B; and 40–43 min, 0% B. A flow rate of 1 mL/min was used [20].

### 5.4. Soil Samples Collection and Analysis

In each population, two samples of soil with a volume of 0.5 dm^3^ were collected. Samples were taken from a depth of over 5 cm in randomly chosen points within the population borders, oven-dried at 50 °C, ground manually with a mortar, and sieved (<0.25 mm sieve). Analyses of calcium (Ca) and iron (Fe) in soils (good indicators of soil acidity, weathering processes, and soil anoxia) were performed after mineralization in 10 mL of 65% HNO_3_ (Ultranal) in a SpeedWave 8212JN microwave mineralizer. Measurements were performed using an atomic absorption spectrometer contrAA 700.

### 5.5. Biomass Sample Collection and Analysis

Since biomass parameters are a good predictor of environmental resources, we assessed habitat quality (fertility) by collecting two samples of plant biomass from a randomly selected 0.25 m^2^ area within the population borders (according to Kotowski and van Diggelen [83]). Biomass samples were oven-dried at 50 °C and pulverized in an automatic mill. In biomass samples, nitrogen (N), phosphorus (P), potassium (K), and carbon (C) contents were measured as follows: total N and total C with a Thermo Scientific Flash2000 CHNS/O Analyzer; total P using a San++ Continuous Flow Analyzer after mineralization in HNO_3_; and total K extracted with ammonium acetate and measured with a flame atomic absorption spectrometer.

### 5.6. Insect Visitors

To determine the taxonomic composition and frequency of insects visiting flowers, we applied the method of Zych [24], that is, we randomly chose a patch of flowering plants, usually consisting of 2–5 shoots in full bloom. Then, using digital cameras (HC-VX870; Panasonic Corporation), which were set on a tripod approximately 1.0–1.5 m from the plants, we recorded insects visiting flowers for 15 min on the chosen patches (if possible for each recording, we chose a different patch). In each of the selected populations, we made 12 recordings of insect activity for at least 2 d between 10.00 and 16.00 (during the highest activity period of insects). In the laboratory, recorded insects visiting flowers were analyzed for the number of visits to flowers and classified into one of the eight following groups: (i) *Apis mellifera*, (ii) *Bombus* spp., (iii) solitary bees, (iv) Syrphidae, (v) Diptera, (vi) Lepidoptera, (vii) Coleoptera, and (viii) other. The frequency of insect visits was calculated per census (15 min) and converted using the number of inflorescences in a recorded patch.

### 5.7. Data Analysis

For each population, we determined the total concentration of AAs, percentage of individual detected amino acids, proportions of non-protein amino acids (NPAAs), protein amino acids (PAAs), and essential amino acids (EAAs) for honeybees (as well as other pollinators): phenylalanine, leucine, arginine, threonine, lysine, isoleucine, valine, methionine, histidine, and tryptophan [28,29].

We used R.3.5.3 and Statistica 13.3 for statistical analyses. Before statistical tests, the data were checked for normality (Shapiro–Wilk test), and if necessary, data were log- or square root-transformed to a normal distribution. To determine the differences between populations and regions, into which the populations were clustered according to their geographical localization, we applied a one-way ANOVA or Kruskal–Wallis ANOVA (for data not normally distributed). This analysis was performed to determine the differences in nectar traits (total sugars and AA concentration, sugar ratios (r), the percentage of sugars, AAs selected for linear mixed models, essential AAs (EAAs), the content of elements in soil and biomass, and the frequency of insects visiting flowers). A one-way ANOVA was applied to determine differences in the percentage share of glucose, fructose, sucrose, glutamine, glutamic acid, serine, phenylanine, isoleucine, EAAs, and sugar ratios as well as N, P, K, Fe, and Ca content. The Kruskal–Wallis ANOVA was applied to determine differences in total sugar and AA concentration, percentage of maltose, lactose, norvaline, and the frequency of insect visits as well as C content. Then, a pairwise t-test was used to calculate pairwise comparisons between group levels with the Benjamini–Hochberg correction for multiple testing. A *p*-value < 0.05 was considered statistically significant.

To establish the significant differences between nectar samples collected from flowers in different sexual phases, a t-test (Student’s t-test) was used to determine sugar ratios (r) and EAA percentage, whereas the non-parametic U-Mann–Whitney test was used to determine total sugar and AA concentration.

We visualized how the three regions were clustered in relation to the nectar composition (AA and sugar percentage) by non-metric multidimensional scaling (NMDS) based on the Bray–Curtis distances, using the R package vegan function “metaMDS”. The significance of separation was tested using the permutational multivariate analysis of variance (PERMANOVA).

To examine the effect of soil conditions and biomass parameters on nectar characteristics, we built linear mixed models (LMM) using the R package caret. To reduce the dimensions of the AA dataset, recursive feature elimination (RFE) was performed [84]. The RFE worked by recursively removing AAs and building a model on those that remained. The model accuracy was used to identify which AAs contributed the most to the prediction of the frequency of insect visits. We chose six AAs, which may be the most important to *P. caeruleum* ecology, from which three were characterized by the highest percentage, and three had the highest influence on the frequency of insect visits. Sugar and AA concentrations, all sugars, and selected AA proportions were used as the dependent variables. Variables expressed as relative proportions were arcsine transformed to meet the model assumptions. Total P, C, K, N, Fe, and Ca of each population were added as fixed factors in these models. The population was included as a random factor (Table 3). For this model, we used the percentage content (proportion) of AAs and sugars to reduce problems regarding concentration changes caused by variable weather conditions and because, as suggested, the composition was much less variable than the concentrations [14,15,39].

Correlations between nectar traits and habitat parameters that were not included in the LMM models, such as population size and frequency of insect visits (including individual taxa), were calculated using Spearman’s correlation.

## Figures and Tables

**Table 1:** Means of the total sugar and amino acid (AA) concentration, sucrose/hexose ratio (r), sugars, and AAs selected for the model, and essential AAs (EAAs) of *Polemonium caeruleum* nectar for 14 studied populations. Pop (population); GLN (glutamine), GLU (glutamic acid), SER (serine), ILE (isoleucine), PHE (phenylalanine), NVA (norvaline). Within a column, two values with a different letter are significantly different at *p* < 0.05; *n* = 2 for each population (except BOB, where *n* = 3).

Pop	Total Sugar [µg/µL]	Total AAs [pmol/µL]	r = S/(F + G)	Fructose [%]	Glucose [%]	Sucrose [%]	Maltose [%]	Lactose [%]	GLN [%]	GLU [%]	SER [%]	ILE [%]	PHE [%]	NVA [%]	% of EAAs
BIA	200.13 b	973.1	0.28	50.63	20.44	19.80	6.11 ab	3.02	10.26 defg	21.45 ab	5.17	2.01	4.82 bc	0.39	24.12 ab
BOB	157.94 b	252.6	0.20	49.14	26.60	15.12	6.61 a	2.53	5.32 g	16.95 bcd	11.37	2.40	3.20 cdef	0.20	16.36 bcde
CZL	145.72 b	110.2	0.74	36.50	18.94	41.05	1.94 ab	1.57	11.44 cdefg	10.73 de	8.51	2.43	6.93 ab	0.00	24.36 ab
DRO	479.81 ab	461.5	1.09	27.93	19.04	51.32	0.90 ab	0.81	17.25 abcde	12.64 cde	11.64	1.59	1.76 fg	0.33	10.25 de
KCZ	276.20 b	474.8	1.55	19.30	19.93	60.77	0.00 b	0.00	11.21 cdefg	11.74 cde	6.91	2.29	4.14 bcde	0.00	22.34 bc
KLE	201.58 b	216.3	1.86	20.25	14.77	64.98	0.00 b	0.00	24.02 abc	14.21 cde	8.52	1.86	2.43 def	1.05	16.42 bcde
KOP	266.10 b	117.7	0.66	33.92	22.22	37.23	3.46 ab	3.17	6.42 efg	18.61 abc	9.28	1.39	4.10 bcde	0.00	20.20 bcd
MAL	149.26 b	132.2	0.07	53.58	28.99	5.51	7.39 a	4.53	5.82 fg	14.37 cde	8.48	2.44	4.73 bcd	0.00	26.88 ab
ORZ	1060.51 a	328.8	1.03	26.32	23.00	50.68	0.00 b	0.00	30.45 ab	13.81 cde	5.32	2.31	4.01 bcde	1.19	16.42 bcde
ROS	522.12 ab	3416.3	0.63	35.73	20.02	34.87	4.50 ab	4.88	29.17 ab	23.34 a	10.79	0.47	0.81 g	0.17	8.38 e
SIE	527.58 ab	793.4	1.02	27.81	20.51	49.36	0.79 ab	1.53	15.88 bcdef	7.87 e	11.83	3.23	2.92 cdef	0.00	11.89 cde
SPN	290.64 b	133.2	0.72	30.95	26.55	41.33	0.75 ab	0.42	5.33 fg	8.17 e	9.19	2.95	3.62 cde	0.00	22.60 bc
WPN	484.96 ab	200.5	2.24	17.51	13.32	69.17	0.00 b	0.00	19.76 abcd	15.83 bcd	6.55	1.35	9.07 a	0.00	39.46 a
ZED	1102.71 a	823.1	1.61	21.97	16.19	61.43	0.38 ab	0.03	33.49 a	12.37 cde	10.60	1.76	2.43 efg	0.00	15.28 bcde
mean	409.9 ± 344.8	590.3 ± 1006	1.1 ± 0.9	32.8 ± 14.4	21.0 ± 7.1	42.1 ± 22.7	2.5 ± 3.2	1.6 ± 2.1	15.8 ± 10.4	14.5 ± 4.8	9.0 ± 3.1	2.0 ± 0.7	3.9 ± 2.2	0.2 ± 0.4	19.5 ± 8.5
*p*	0.043	ns	0.040	ns	ns	0.023	0.025	ns	0.000	0.000	ns	ns	0.000	ns	0.046

**Table 2:** Nectar characteristics during male (M) and female (F) sexual phases of *Polemonium caeruleum* flowers. Total sugar and amino acid (AA) concentration, r (sucrose/hexose ratio), percentage of three main sugars (fructose, glucose, and sucrose), and essential amino acids (EAAs: phenylalanine, leucine, arginine, threonine, lysine, isoleucine, valine, methionine, histidine, and tryptophan). Data represent mean values for nectar obtained from flowers in male and female sexual phases, from 14 populations ± SD, *n* = 15 for the male phase, *n* = 14 for the female phase. Significant differences between M and F sexual phases are marked with * at *p* < 0.05.

Sex Phase	Total Sugar [µg/µL]	Total AAs [pmol/µL]	r = S/(F + G)	Fructose [%]	Glucose [%]	Sucrose [%]	EAAs [%]
M	400.9 ± 403.4	762.6 ± 1325	1.10 ± 0.5 *	28.23 ± 14.2	17.96 ± 7.7 *	50.34 ± 22.3 *	17.70 ± 7.2
F	419.7 ± 283.8	405.7 ± 467.4	0.73 ± 0.4 *	37.8 ± 13.4	24.16 ± 4.7 *	33.23 ± 20.2 *	21.48 ± 9.5

**Table 3:** Results of the linear mixed models performed to examine the effect of biomass and soil nutrients on nectar characteristics of *Polemonium caeruleum*; TC (total carbon), TN (total nitrogen), TK (total potassium), TP (total phosphorus); estimates, standard error. * 0.05 ≤ *p* < 0.1; ** 0.001 ≤ *p* < 0.05. AA (amino acids), GLN (glutamine), GLU (glutamic acid), SER (serine), ILE (isoleucine), PHE (phenylalanine), and NVA (norvaline). The size of the population was included as a random factor.

	TC Biomass		TN Biomass		TK Biomass		TP Biomass		Ca Soil		Fe Soil	
	Estimate	Std. Error	Estimate	Std. Error	Estimate	Std. Error	Estimate	Std. Error	Estimate	Std. Error	Estimate	Std. Error
Total sugar [ug/uL]	57.406	50.168	−90.250	122.435	94.622	113.410	1872.687	991.600	−53.469	153.980	−12.357	49.731
Fructose [%]	−0.555	2.693	0.781	5.976	4.048	6.006	16.026	48.742	3.136	7.387	−1.367	2.258
Glucose [%]	−0.054	1.477	0.171	2.785	0.281	3.190	−7.378	22.373	3.744	3.344	−1.240	0.937
Sucrose [%]	0.274	4.007	−2.075	9.169	−7.747	8.977	−12.987	74.689	−7.099	11.394	2.320	3.542
Maltose [%]	−0.00001	0.005	0.009	0.012	0.012	0.012	0.020	0.100	0.002	0.015	0.001	0.005
Lactose [%]	−0.003	0.004	0.008	0.008	0.006	0.009	−0.061	0.068	−0.005	0.010	0.003	0.003
Total AAs [pmol/uL]	−137.005	183.533	−357.063	366.971	−380.308	401.647	−2436.125	2978.642	−684.877	445.451	289.963 *	128.893
GLN [%]	0.006	0.014	0.017	0.034	−0.037	0.031	0.188	0.274	−0.055	0.043	0.023	0.014
GLU [%]	−0.0003	0.006	−0.004	0.016	0.005	0.015	−0.111	0.131	−0.037	0.021	0.006	0.007
SER [%]	−0.004	0.006	0.006	0.011	−0.026 *	0.013	−0.200 *	0.089	−0.028 *	0.013	0.007	0.004
PHE [%]	−0.004	0.003	0.003	0.007	−0.001	0.007	−0.071	0.060	−0.005	0.009	−0.002	0.003
ILE [%]	0.0003	0.001	−0.001	0.003	−0.006	0.003	0.003	0.025	0.003	0.004	−0.002	0.001
NVA [%]	−0.0003	0.0005	−0.001	0.001	−0.003 **	0.001	0.022 *	0.010	−0.0003	0.002	−0.00004	0.001

**Table 4:** Correlations between nectar traits and habitat parameters with variables that were not included in the LMM models. TRP (tryptophan), LYS (lysine), NVA (norvaline), GLY (glycine), ALA (alanine), BABA (β-alanine), TYR (tyrosine), AABA (α-aminobutyric acid), VAL (valine). Table contains only significant results (*p* < 0.05).

Trait 1	Trait 2	r
Frequency of visits by *Bombus* spp.	TRP [%]	−0.80
	LYS [%]	−0.79
Frequency of visits by *A. mellifera*	NVA [%]	0.81
Frequency of visits by Syrphidae	glucose [%]	0.85
	Total AAs [pmol/uL]	−0.78
	GLY [%]	0.85
Frequency of visits by Lepidoptera	glucose [%]	0.93
	Total AAs [pmol/uL]	−0.93
	ALA [%]	0.92
	BABA [%]	0.82
	TYR [%]	0.85
	AABA [%]	−0.86
	VAL [%]	0.85

## Supplementary Materials

The following are available online at https://www.mdpi.com/2223-7747/9/10/1297/s1, Table S1: Sizes and distribution of investigated populations of *Polemonium caeruleum*, Table S2: Mean content of elements selected for analysis in soil and biomass, Table S3: Percentage composition of amino acids (AAs) in the nectar of *Polemonium caeruleum*.
