# Supplementary material for: Intraspecific Variation in Nectar Chemistry and Its Implications for Insect Visitors: The Case of the Medicinal Plant, Polemonium Caeruleum L
Source: Plants (Basel). 2020 Oct 1;9(10):1297. doi: 10.3390/plants9101297 (PMC7600102; doi:10.3390/plants9101297)
Supplement: Supplementary file 1 [file plants-09-01297-s001.pdf]

Table S1. Percentage composition of amino acids (AAs) in the nectar of *Polemonium caeruleum*. The essential AAs are marked with bold font, the non-protein AAs are underlined, ASP – Asparagine, GLU - Glutamic acid, ASN – Asparagine, SER – Serine, GLN – Glutamine, HIS - Histidine, GLY – Glycine, THR – Threonine, CIT – Citrulline, ARG – Arginine, BALA – β – Alanine, ALA – Alanine, TAU – Taurine, GABA -γ -Aminobutyric Acid, BABA - β-Aminobutyric acid, TYR – Tyrosine, AABA - α-Aminobutyric acid, CYS – Cystine, VAL – Valine, MET – Methionine, NVA – Norvaline, TRP – Tryptophan, PHE – Phenylalanine, ILE – Isoleucine, ORN – Ornithine, LEU – Leucine, LYS – Lysine, SAR – Sarcosine, and PHE – Phenylalanine. Number of nectar samples was n = 2 for each population (except BOB, where n = 3).

Population code	ASP	GLU	ASN	SER	GLN	**HIS**	GLY	**THR**	CIT	**ARG**	BALA	ALA	TAU	GABA	BABA	TYR	AABA	CYS	**VAL**	**MET**	NVA	**TRP**	**PHE**	**ILE**	ORN	**LEU**	**LYS**	SAR	PRO
BIA	3.7	21.4	1.8	5.2	10.3	0.3	8.0	2.6	0.7	2.6	10.5	0.0	0.8	0.0	2.1	0.8	1.5	2.3	0.7	1.4	0.4	0.3	4.8	2.0	3.3	2.4	7.2	0.6	2.3
BOB	5.5	17.0	2.1	11.4	5.3	0.5	9.3	2.5	1.2	2.9	9.3	3.8	0.7	0.0	3.9	2.2	0.0	2.5	1.7	0.6	0.2	0.0	3.2	2.4	2.7	2.1	0.5	1.7	4.8
CZL	5.5	10.8	3.1	8.5	11.5	0.4	4.1	6.9	0.4	3.6	7.6	11.8	0.0	0.0	3.3	3.8	0.0	2.3	0.0	0.0	0.0	0.0	6.9	2.4	1.8	4.1	0.0	0.0	1.2
DRO	6.2	12.5	2.7	11.6	17.2	0.1	5.9	2.9	1.5	2.0	7.1	2.0	0.9	0.0	8.5	2.4	0.0	1.9	0.6	0.6	0.3	0.0	1.8	1.6	2.5	0.0	0.7	0.0	6.5
KCZ	4.5	11.8	1.6	6.9	11.3	0.0	7.2	2.5	1.3	1.5	7.8	9.2	0.3	0.0	3.1	2.7	0.0	2.1	0.5	0.9	0.0	0.0	4.1	2.3	3.0	2.0	8.4	0.3	4.7
KLE	4.3	14.2	2.1	8.5	24.0	0.2	6.7	2.4	1.4	2.6	6.3	3.9	0.5	0.0	3.3	2.3	0.0	1.6	0.7	1.3	1.0	0.0	2.4	1.9	3.5	3.0	1.9	0.0	0.0
KOP	5.6	18.6	2.8	9.3	6.4	0.0	7.3	2.3	1.9	3.9	9.5	9.0	0.0	0.0	2.9	3.2	0.0	3.4	2.9	0.7	0.0	0.0	4.1	1.4	0.0	3.4	1.4	0.0	0.0
MAL	5.0	14.5	2.0	8.5	5.8	0.5	7.1	2.9	1.7	7.1	7.1	6.1	0.0	1.5	3.8	2.9	1.8	1.7	1.2	1.4	0.0	0.0	4.7	2.4	3.7	0.9	5.7	0.0	0.0
ORZ	2.9	13.8	1.7	5.3	30.3	0.8	6.1	1.7	0.4	2.1	4.9	0.7	0.8	0.0	7.3	1.5	0.0	1.5	0.8	0.6	1.2	0.0	4.0	2.3	5.1	1.9	2.3	0.0	0.0
ROS	4.6	23.3	1.6	10.8	29.2	0.1	2.5	2.6	0.2	2.3	12.5	0.0	2.4	0.0	2.4	0.3	0.4	1.2	0.0	0.7	0.2	0.0	0.8	0.5	0.0	1.1	0.3	0.0	0.0
SIE	5.8	7.9	2.8	11.8	15.8	0.2	5.4	2.8	2.2	2.8	6.9	3.7	0.0	0.0	8.2	3.6	0.0	2.0	0.0	0.0	0.0	0.0	2.9	3.2	8.9	0.0	0.0	0.0	3.1
SPN	3.4	8.2	2.2	9.2	5.3	0.4	8.7	2.5	1.3	2.9	11.1	4.2	0.0	0.0	12.5	4.1	0.0	2.4	9.9	0.0	0.0	0.0	3.6	2.9	4.8	0.0	0.4	0.0	0.0
WPN	3.2	15.8	0.5	6.5	19.9	0.6	2.8	3.3	1.2	1.8	3.4	1.6	0.0	0.0	3.1	1.6	0.0	0.9	1.8	1.3	0.0	0.0	9.1	1.4	0.0	19.6	0.6	0.0	0.0
ZED	3.9	12.4	2.1	10.6	33.4	0.6	1.6	3.5	1.2	2.7	8.8	0.0	0.0	0.0	2.3	1.4	0.4	2.0	0.0	0.0	0.0	1.5	2.4	1.8	4.6	1.2	1.6	0.0	0.0

Table S2. Mean content of elements selected for analysis in soil and biomass. Results of one-way ANOVA for normally distributed data, or Kruskal-Wallis ANOVA followed by a pairwise t-test comparisons between group levels with Benjamini-Hochberg correction for multiple testing, bars sharing common letters do not have significant differences (p-value > 0.05).

Population code	Fe %	Ca %	TK %	TN%	TC%	TP%
BIA	0.65<br>de	0.10<br>d	1.71	1.72<br>bc	42.95	0.26<br>a
BOB	1.65<br>cd	0.51<br>c	1.62	1.69<br>bc	42.66	0.14<br>a
CZL	3.60<br>b	1.01<br>b	1.27	2.04<br>bc	42.49	0.19<br>a
DRO	2.30<br>cd	0.10<br>d	0.71	2.87<br>ab	42.43	0.33<br>a
KCZ	4.93<br>ab	1.56<br>ab	1.70	2.15<br>bc	41.99	0.22<br>a
KLE	5.08<br>ab	1.30<br>ab	1.49	2.73<br>ab	41.04	0.30<br>a
KOP	0.82<br>d	0.17<br>d	1.80	2.77<br>ab	41.25	0.19<br>a
MAL	2.50<br>bc	1.61<br>ab	0.91	4.01<br>a	40.14	0.25<br>a
ORZ	1.12<br>d	0.46<br>c	1.01	2.29<br>bc	43.84	0.25<br>a
ROS	5.79<br>a	1.12<br>b	1.36	1.77<br>bc	42.62	0.12<br>a
SIE	1.14<br>d	0.14<br>d	1.49	2.35<br>bc	42.96	0.14<br>a
SPN	0.24<br>e	1.77<br>a	0.81	1.50<br>c	42.10	0.09<br>a
WPN	0.52<br>e	0.29<br>cd	1.86	2.52<br>abc	42.16	0.15<br>a
ZED	1.36<br>d	0.23<br>cd	1.05	2.51<br>abc	44.28	0.24<br>a
mean	2.18±1.8	0.85±0.76	1.3±0.53	2.35±0.68	42.39±1.34	0.2±0.08
p	0.02	0.000	ns	0.03	ns	0.047

Table S3. Sizes and distribution of investigated populations of *Polemonium caeruleum*.

Region (voivodeship, geographical localization)	Geographical coordinates	Population code	No. of flowering shoots
Podlaskie, NE	52°41'20" N<br>23°52'42" E	BIA	100
West Pomeranian, N	53°57'46" N<br>16°34'24" E	BOB	40
Świętokrzyskie, S	50°35'45" N<br>19°51'46" E	CZL	500

Mazovian, NE	52°33'07" N<br>22°27'36" E	DRO	600
Pomeranian, N	54°22'44" N<br>18°19'13" E	KCZ	700
Mazovian, NE	53°02'55" N<br>21°51'41" E	KLE	1100
Podlaskie, NE	53°15'55" N<br>22°36'38" E	KOP	100
Świętokrzyskie, S	50°49'36" N<br>20°18'19" E	MAL	6,000
Podlaskie, NE	52°40'47" N<br>23°31'47" E	ORZ	10,000
Podlaskie, NE	53°54'40" N<br>22°56'17" E	ROS	120
Podlaskie, NE	52°53'51" N<br>23°53'34" E	SIE	35
Pomeranian, N	54°42'42" N<br>17°26'32" E	SPN	70
Podlaskie, NE	54°07'06" N<br>23°04'28" E	WPN	450
Podlaskie, NE	53°06'36" N<br>23°27'38" E	ZED	15,000
